# A difficult‐to‐diagnose case of Borrmann type IV gastric cancer with marked transverse colon stenosis

**DOI:** 10.1002/jgh3.12832

**Published:** 2022-10-20

**Authors:** Kazuki Natsui, Masaki Maruyama, Shuji Terai

**Affiliations:** ^1^ Department of Gastroenterology Kashiwazaki General Hospital and Medical Center Kashiwazaki Niigata Japan; ^2^ Division of Gastroenterology and Hepatology Graduate School of Medical and Dental Sciences, Niigata University Chuo‐Ku Niigata Japan

**Keywords:** colon stenosis, colonic metastasis, gastric cancer, peritoneal dissemination

## Abstract

We report a difficult‐to‐diagnose case of Borrmann type IV gastric cancer with poor typical findings and marked colonic stenosis in a 47‐year‐old man and present a literature review.

## Graphical abstract

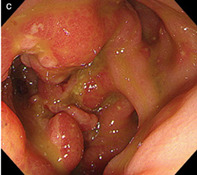

## Introduction

Advanced gastric cancer usually presents with epigastric or bleeding symptoms from the tumor such as black stools, hematemesis, and iron deficiency anemia. Advanced gastric cancer usually presents as an elevated lesion and is often detected on computed tomography (CT) or esophagogastroduodenoscopy (EGD). We report a difficult‐to‐diagnose case of Borrmann type IV gastric cancer with poor typical findings and marked colonic stenosis.

## Case report

**Figure 1:**
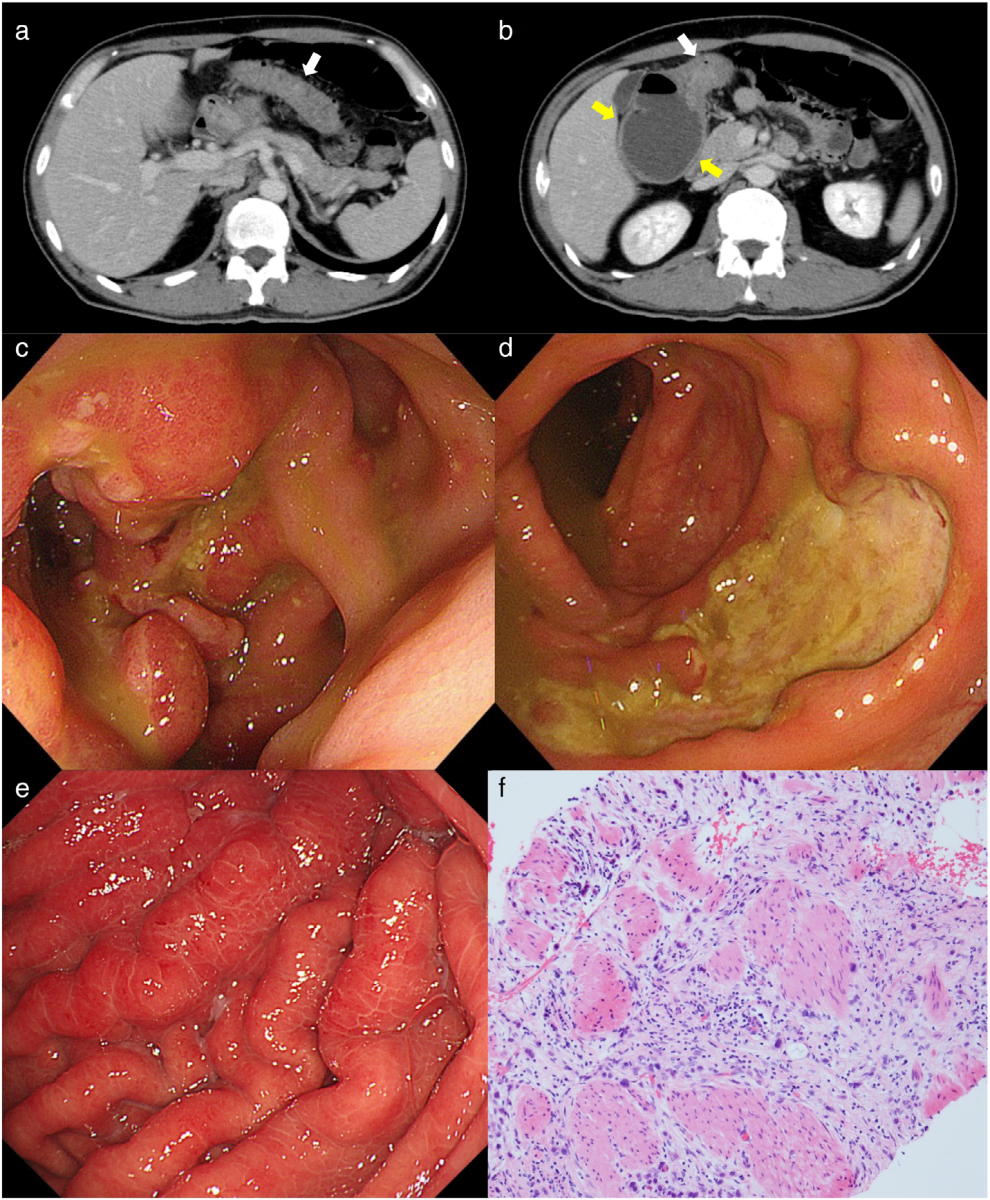
(a and b) Computed tomography showed an approximately 13‐cm long contrast‐enhanced edematous wall thickening of the transverse colon (white arrows) and an alarming rupture‐like dilatation and intestinal effusion from the ascending colon to the terminal ileum (yellow arrows). (c and d) Colonoscopy showed mild stenosis of the transverse colon and punched‐out multiple ulcers in the ascending colon. (e) Esophagogastroduodenoscopy showed poorly dilating gastric wall thickening of the antrum and lower gastric body. (f) HE staining showed undifferentiated adenocarcinoma.

A 47‐year‐old man with no past medical history presented with a complaint of colicky pain in the entire abdomen, fever, and watery stools that started about 1 month previously. He had a high fever (38.4°C) and tenderness in the lower abdomen. Laboratory examination on admission revealed signs of acute inflammatory response (elevated white blood cell count, 10 400/μL [normal range 4000–8000/μL] and elevated C‐reactive protein, 19.4 mg/dL, [normal range <0.3 mg/dL]), hypoalbuminemia (albumin 2.8 g/dL [normal range 3.9–4.9 g/dL]), and normal range of serum tumor markers (carcinoembryonic antigen, 2.6 ng/mL [normal range <5 ng/mL] and carbohydrate antigen 19–9, 16 U/mL [normal range <37 U/mL]). CT imaging (Fig. 1a,b) showed an approximately 13‐cm long contrast‐enhanced edematous wall thickening of the transverse colon (white arrows). Additionally, it revealed an alarming rupture‐like dilatation and intestinal effusion from the ascending colon to the terminal ileum (yellow arrows). Colonoscopy showed mild stenosis of the transverse colon and punched‐out multiple ulcers in the ascending colon (Fig. 1c,d). The patient was hospitalized with a diagnosis of transverse colon stenosis and obstructive enteritis/impending rupture of the ascending colon due to any cause. On Day 1, a transanal ileus tube was inserted to prevent the rupture of the ascending colon and reduce intestinal pressure. Antibiotic therapy was ineffective. On Day 16, EGD for cancer screening revealed poorly dilating gastric wall thickening of the antrum and the lower gastric body (Fig. 1e), and the biopsy showed normal gastric mucosa. On Day 20, endoscopic ultrasound‐guided fine‐needle aspiration of the gastric antrum detected undifferentiated adenocarcinoma (HER2‐negative) (Fig. 1f). We made a diagnosis of Borrmann type IV gastric cancer with transverse colon stenosis caused by peritoneal dissemination and obstructive enteritis of the ascending colon. Thereafter, multiple surgical procedures including ileostomy, gastrojejunal bypass, and bilateral ureteral stenting for hydronephrosis were performed. Moreover, multiple chemotherapy treatments including nab‐paclitaxel/ramucirumab, S‐1/cisplatin, and nivolumab were administered, but the patient died about 1.2 years after the initial visit. Autopsy was carried out according to the patient's wish before death. The findings included typical gastric cancer with undifferentiated adenocarcinoma, carcinomatous peritonitis, and peritoneal dissemination, which was consistent with the stenosis of the transverse colon.

## Discussion

Gastric cancer is the third most common cause of cancer‐related death both globally^1^ and in Japan. The macroscopic appearance of advanced gastric cancers is usually classified with the Borrmann classification into four types,^2^ and patients with type IV gastric cancer typically have the poorest survival.^3^

The initial cause of the transverse colon stenosis in this case was thought to be peritoneal dissemination and direct tumor invasion, based on the autopsy, but the possibility of colonic metastasis of gastric cancer was also raised. According to Su *et al*., colonic metastasis of gastric cancer is extremely rare. They also reported that the colonoscopic findings of the colonic metastasis were only wall thickening and swelling, unlike the classic appearance of primary colorectal cancer, which is contact bleeding or centrally ulcerated lesions in the colon.^4^ Additionally, Jang *et al*. reported most of the patients with gastric cancer with colonic metastasis had poorly differentiated adenocarcinoma.^5^ Their findings were similar to those associated with transverse colon stenosis in our case.

Our patient presented with marked transverse colon stenosis, no epigastric or bleeding symptoms, and normal serum tumor markers, which made the diagnosis difficult. Therefore, when an unexplained stenosis of the transverse colon is found, the possibility of Borrmann type IV gastric cancer should be considered in the diagnostic process.

## References

[1] Smyth EC , Nilsson M , Grabsch HI , van Grieken NCT , Lordick F . Gastric cancer. Lancet. 2020; 396: 635–48. 32861308 10.1016/S0140-6736(20)31288-5

[2] Borrmann R , Henke F , Lubarsch O . Handbuch der Speziellun Pathologischen Anatomie und Histologie, Vol. 4. Berlin: Springer, 1926; 865.

[3] Li C , Oh SJ , Kim S *et al*. Macroscopic Borrmann type as a simple prognostic indicator in patients with advanced gastric cancer. Oncology. 2009; 77: 197–204. 19729977 10.1159/000236018

[4] Su WC , Tsai HL , Wu CC *et al*. Two rare cases of synchronous and metachronous colonic metastases in patients with advanced gastric cancer. World J. Surg. Oncol. 2018; 16: 21. 29386011 10.1186/s12957-018-1323-8 PMC5793428

[5] Jang HJ , Lim HK , Kim HS *et al*. Intestinal metastases from gastric adenocarcinoma: helical CT findings. J. Comput. Assist. Tomogr. 2001; 25: 61–7. 11176295 10.1097/00004728-200101000-00011

